# Thermogenesis-triggered seed dispersal in dwarf mistletoe

**DOI:** 10.1038/ncomms7262

**Published:** 2015-02-09

**Authors:** Rolena A. J. deBruyn, Mark Paetkau, Kelly A. Ross, David V. Godfrey, John S. Church, Cynthia Ross Friedman

**Affiliations:** 1 Department of Biological Sciences, Thompson Rivers University, Kamloops, British Columbia, Canada V2C 0C8; 2 Department of Physical Sciences (Physics), Thompson Rivers University, Kamloops, British Columbia, Canada V2C 0C8; 3 Pacific Agri-Food Research Centre, Agriculture and Agri-Food Canada, 4200 Highway 97, Summerland, British Columbia, Canada V0H 1Z0; 4 Department of Natural Resource Science, Thompson Rivers University,, Kamloops, British Columbia, Canada V2C 0C8

## Abstract

Lodgepole pine dwarf mistletoe (DM), *Arceuthobium americanum*, is a parasitic flowering plant and forest pathogen in North America. Seed dispersal in DM occurs by explosive discharge. Notably, slight warming of ripe DM fruit in the laboratory can trigger explosions. Previously, we showed that alternative oxidase, a protein involved in endogenous heat production (thermogenesis) in plants, is present in DM fruit. These observations have led us to investigate if thermogenesis induces discharge. Here, infrared thermographs reveal that ripe DM fruits display an anomalous increase in surface temperature by an average of 2.1±0.8 °C over an average time of 103±29 s (*n*=9, 95% confidence interval) before dehiscence. Furthermore, both non-isothermal and isothermal modulated differential scanning calorimetry consistently show an exothermic event (~1 J g^−1^) in the non-reversible heat flow just prior to discharge. These results support thermogenesis-triggered seed discharge, never before observed in any plant.

## Abstract (web summary)

Lodgepole pine dwarf mistletoe is a parasitic forest pathogen that disperses its seeds via an explosive discharge. Here, deBruyn *et al*. show that ripened mistletoe fruits generate heat less than two minutes before this discharge, suggesting a role for thermogenesis in seed dispersal.

## Graphical abstract

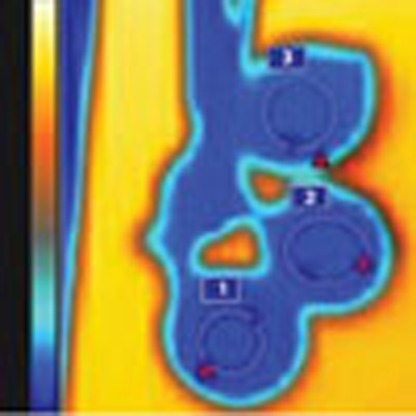

Dwarf mistletoes (DMs) belong to the genus *Arceuthobium* (family Santalaceae), and comprise a group of new and old world flowering plants that are aerial parasites on Pinaceae and Cupressaceae^1^. These parasites are economically important in Canada, especially in British Columbia (BC) and Alberta, where they are destructive pathogens of commercially valuable coniferous timber trees. Infestation by DM is detrimental to host wood quality, impedes the trees’ ability to resist other infestation and decreases the lifespan of infected trees. Currently, six *Arceuthobium* taxa have ranges that extend over nearly three quarters of BC, equating to a potential loss of >3.7 million m^3^ of coniferous forest a year^2^. *Arceuthobium americanum* Nutt. ex Engelm., the American or lodgepole pine DM, commonly infects lodgepole pine (*Pinus contorta* Dougl. ex Loud. var. *latifolia* Engelm.) in BC, where it is of considerable concern to the forest industry in that province.

Seed dispersal in DM is accomplished by a remarkable explosive process. DM spread solely by seed, which is forcibly ejected from the ripe single-seeded fruit at the end of the growing season^1^ (late August to early September in BC). For a given stand of infected pines, dispersal takes place over the period of about a week. Ripe fruit are broadly fusiform-spheric, bicoloured and borne on short, recurved pedicels in groups of three or more on branching infructescences (Fig. 1). Seminal studies by Hinds *et al*.^3^ showed that a seed can be dispersed as far as 20 m from its source, with initial velocities approaching 25 m s^−1^ (100 km h^−1^) . Viscin tissue, a mucilaginous region that forms a layer around the single seed within each fruit, accumulates the hydrostatic force needed for water-driven explosive discharge^1^,^4^. Dispersal is ultimately achieved by explosive fracture in the abscission layer at the pedicel^3^,^4^,^5^. A somewhat analogous strategy for seed dispersal has been described for other plants, such as *Ecballium elaterium* (L.) A. Rich. (Mediterranean squirting cucumber), in which a hydrostatic pressure is also generated within the fruit^6^. *Hura crepitans* L. (Monkey’s dinner bell) similarly undergoes explosive fracture and water-related movement during seed dispersal, although differential drying rather than hydrostatic pressure drives the discharge in this species^7^.

To study explosive discharge of DM fruit in the laboratory, Hinds *et al*.^3^ were able to initiate dehiscence by slightly warming ripe fruit. While we cannot guess what these authors might have thought about the phenomenon, perhaps like us, they began to wonder if endogenous heat production (thermogenesis) might play a role in explosive discharge. Thermogenesis is the internal maintenance or increase of tissue temperature^8^,^9^,^10^. In plants, thermogenesis is most often accomplished through mitochondrial respiratory pathways that involve a cyanide-resistant alternative oxidase (AOX)^11^. In contrast to the familiar respiration pathways that store energy by creating a proton gradient to generate ATP, AOX pathways create energy and release it as heat^9^. Ross Friedman *et al*.^12^ found that the timing of explosive seed discharge in DM corresponds to the appearance of AOX in DM fruit mitochondria, which suggests that if there is a proper supply of substrate (for example, reduced ubiquinone) and a stable metabolic flow towards the mitochondria (including a supply of NADH), thermogenesis might induce seed dispersal.

Some flowering plants are known to employ thermogenesis for reproduction. For example, it is widely believed that the heat produced by thermogenesis in some Araceae volatilizes chemicals that attract pollinators to the flowers^9^. It is, therefore, not unreasonable to think that thermogenesis might play other roles in reproduction, such as in seed dispersal.

In our current study, infrared thermography (IRT) along with both non-isothermal (ramped temperature) and isothermal (constant temperature) modulated differential scanning calorimetry (MDSC) is used to detect endogenous heat production in ripe DM fruit before dehiscence to see if thermogenesis could indeed be occurring and possibly even inducing discharge. An average endogenous heat release of ~1 J g^−1^ via MDSC and an associated surface temperature increase of ~2 °C from IRT in the minutes leading up to dispersal is identified in this study, supporting our hypothesis that thermogenesis may play a key role in explosive seed discharge in DM.

## Results

### Infrared thermography

**Figure 2:**
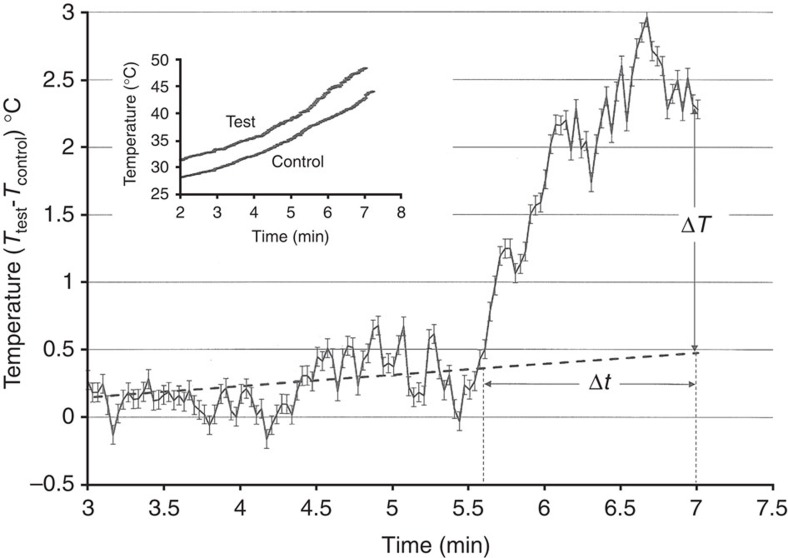
Time series plot of temperature difference (Δ*T=T*_test_−*T*_control_). In these representative data, dehiscence occurs near 7 min; 90 s prior, the temperature of the fruit begins to increase more rapidly than the control. The straight dashed line is the best linear fit to the data prior to 5.5 min, and extrapolation of this line defines Δ*T* and Δ*t*. For this exploding (test) fruit, Δ*T*~2.2 °C and Δ*t*~90 s. The inset (upper left) shows the raw data of the exploding and control fruit. The main graph has been shifted down 3.2 °C for clarity. Here error bars (tolerance) are for temperature (±0.05 °C), and are obtained from the infrared camera’s temperature sensitivity of ≤0.05 °C. This sample is one of nine trials.

All ripe test fruit examined (*n*=9) with IRT displayed an anomalous positive surface temperature increase that initiated a short time (Δ*t*) before dehiscence. An example of the anomalous increase in fruit surface temperature can be seen in Fig. 2, which depicts the time series of the temperature difference *T*_test_−*T*_control_. Early in the time series a linear increase is noted, indicating the test fruit is heating slightly faster than the control. In a perfect experiment, the control and test fruits would be identical in shape and size, and thus heat at the same rate so that the difference *T*_test_−*T*_control_ would be constant; however, no two fruit can be identically shaped or sized, and so there is a slight difference in the rates (see raw data in Fig. 2 inset) leading to a non-zero slope in the time series before 5.5 min. In the event shown in Fig. 2, the temperature difference anomaly starts at about 5.5 min; then, 1.5 min later, dehiscence occurs. By fitting the data from the 3- to 5.5-min period to a linear trend line, the temperature increase at dehiscence (Δ*T*) and the duration time of the anomaly (Δ*t*) can be measured and used to describe the anomaly; that is, the anomaly results in an increase in temperature of Δ*T* and it commences Δ*t* before dehiscence. The values and 95% confidence intervals (*n*=9) for these two parameters are: Δ*T*=2.1±0.8 °C and Δ*t*=103±29 s. The anomalous temperature increase occurring just before dehiscence is evidence of thermogenesis. It is reasonable to expect that DM should undergo some ambient heating in the field similar to that which it experienced during the IRT experiments: plant leaf surface temperatures as high as 51 °C can be reached during a sunny day^13^. The existence of ambient heating, though, does not negate the evidence for thermogenesis.

### Modulated differential scanning calorimetry

**Figure 3:**
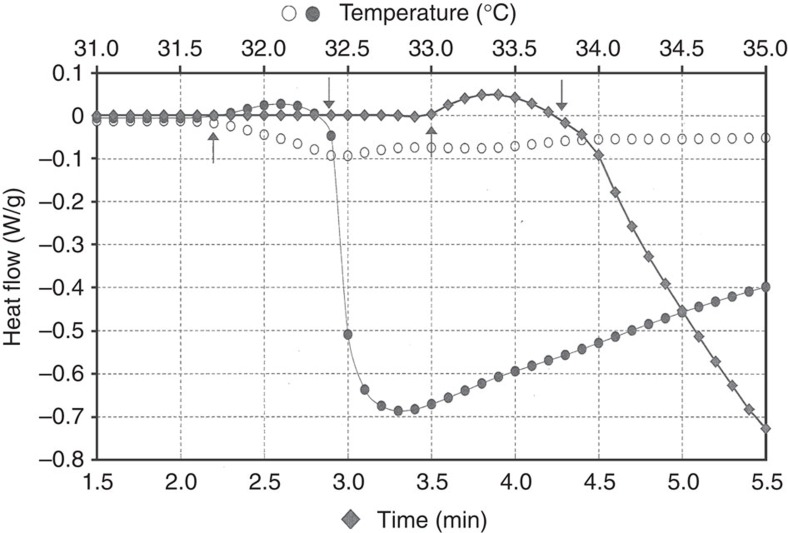
Representative MDSC data. Circles represent non-isothermal (scanning) data and correspond to the upper horizontal scale, temperature in °C). The open circles (○) are reversible heat flow data and the filled circles ([cirf ]) are non-reversible heat flow data. The non-isothermal heat flows show local minima at about 32.4 °C (down arrow), which are associated with dehiscence, and the dips in both data suggest a large endothermic event. Just before dehiscence, however, there is a small positive peak in the non-reversible heat flow data (starting at the up arrow), which is associated with an exothermic event. The filled diamonds (♦) represent the isothermal data and correspond to the lower horizontal scale, time in minutes. Again there is a small release of energy in the region indicated by the up and down arrows.

Results from both non-isothermal and isothermal MDSC confirm that thermogenesis occurs prior to discharge. Figure 3 depicts the MDSC data and shows a representative non-isothermal run (circles) as well as a representative isothermal run (diamonds). The non-isothermal data correspond to the upper horizontal axis (temperature in °C); the open circles track the reversible heat flow data, while the filled circles show the non-reversible heat flow. In the non-isothermal data, both reversible and non-reversible heat flow is constant until about 31.75 °C; at 31.75 °C, the reversible heat flow begins to decrease (associated with wax and polysaccharide melt, an endothermic event), while the non-reversible heat flow begins to increase (an exothermic event). This increase in the non-reversible heat flow indicates thermogenesis. These trends in both the reversible and non-reversible flow continue until the explosion occurs at 32.4 °C, which is characterized by a minimum in the reversible data and a large drop in the non-reversible data. Again, heating in the field could occur and be similar to that experienced in the non-isothermal DSC experiment, but this ambient heating, though, does not invalidate the consistent observation of thermogenesis.

Isothermal MDSC experiments performed at 25, 37 or 50 °C were used to verify that the thermogenic event would occur in the absence of temperature ramping. All isothermal experiments showed an exothermic event before dehiscence, indicative of thermogenesis. The timing of the onset of exothermic event varied from ~1.2 to 9 min, and no trend between isothermal test temperature and the timing of exothermic event was evident. Dehiscence, noted by the onset of the endothermic event and occurring after the exothermic event, took place from~1.8 to 10 min. Figure 3 depicts a representative thermogram showing non-reversible heat flow (diamonds) under isothermal conditions (*T*=37 °C); isothermal MDSC only reports changes in non-reversible heat flow data. The isothermal data correspond to the lower horizontal axis (time in minutes). The behaviour of the isothermal non-reversible heat flow is very similar to that for the non-isothermal runs. As in the non-isothermal case, the non-reversible heat flow for the isothermal test shown in Fig. 3 remains constant until ~1.2 min, when it then begins to increase (exothermic, thermogenesis). The fact that exothermic events such as the ones depicted in Fig. 3 are evident in data in the isothermal experiments performed at different temperatures (25, 37 and 50 °C) and that the isotherms occur over a range of times shows that the exothermic behaviour is independent of the heating rate and isotherm temperature and thus, the isotherm data confirm that the exothermic event is not an artifact of heating the DM.

### Thermogenesis

Using a nominal pre-explosion specific heat from the MDSC data of 0.6 ±0.4 J °C^−1^ g^−1^ (*n*=9, s.d.) for the DM fruit, the energy per gram required for a temperature increase of *ΔT* ~2 °C as measured by IRT is ~1.2±0.8 J g^−1^ (*n*=9, s.d.). Quantitatively, the exothermic events detected with non-isothermal MDSC experiments released an average heat of 0.77±0.08 J g^−1^, or roughly 1% of the total (endothermic and exothermic) heating, while the exothermic events detected with isothermal MDSC experiments released an average heat of 1.25±0.79 J g^−1^, or roughly 1.5% of the total (endothermic and exothermic) heating. The values obtained through IRT, non-isothermal MDSC, and isothermal MDSC are all within the bounds of experimental uncertainty, providing support for the idea that all of the experiments are examining related events.

## Discussion

Given that the IRT experiments and MDSC experiments approach the measurement of thermogenesis differently—and that non-isothermal and isothermal MDSC are quite different in how they measure heat flow—the fact that we obtained similar values for heat flow supports the assertion that all experiments are measuring a real and consistent thermogenic event. In the field, during the week or so over which dispersal occurs, ambient temperatures can slowly rise throughout the day (as mimicked by our non-isothermal tests), but can also remain constant for hours at a time (as reproduced in our isothermal tests). Therefore, while the thermogenic event manifested under both types of laboratory conditions, we need to do more work in the field to try to determine the link between ambient heating and endogenous warming, if any.

Work by Ross Friedman *et al*.^12^ indicated that AOX in mitochondria show increased expression during fruit development. In many plants, heat is generated in the mitochondria as a secondary process of cellular respiration, and is enabled by electron transport chain AOX^11^. Ross Friedman *et al*.^12^ also thought that plastid terminal oxidases (PTOX), which became labelled with anti-AOX antibodies in maturing fruit plastids, might even have some role in thermogenesis. The possible function of PTOX in thermogenesis—if any—is less clear, but discovery of its involvement in thermogenesis might be novel. Future studies of thermogenesis in DM should endeavour to identify the molecular basis of thermogenesis to determine if there is a stable metabolic flow of substrates towards the mitochondria and/or plastids in DM fruit. These studies should be coupled with those in which AOX and PTOX gene expression and protein synthesis is monitored in DM fruits during their development.

In conclusion, this study provides compelling evidence that thermogenesis triggers explosive seed discharge in DM, a novel finding with respect to seed dispersal. Findings of an average endogenous heat release of ~1 J g^−1^ via MDSC and an associated surface temperature increase of ~2 °C from IRT in the minutes leading up to dispersal support this conclusion. The data show that dehiscence is preceded by a thermogenic event. A logical conclusion is that endogenous heat production acts as an internal trigger for the final explosive fracture of the fruit at the abscission layer in the pedicel.

## Methods

### Infrared thermography

**Figure 4:**
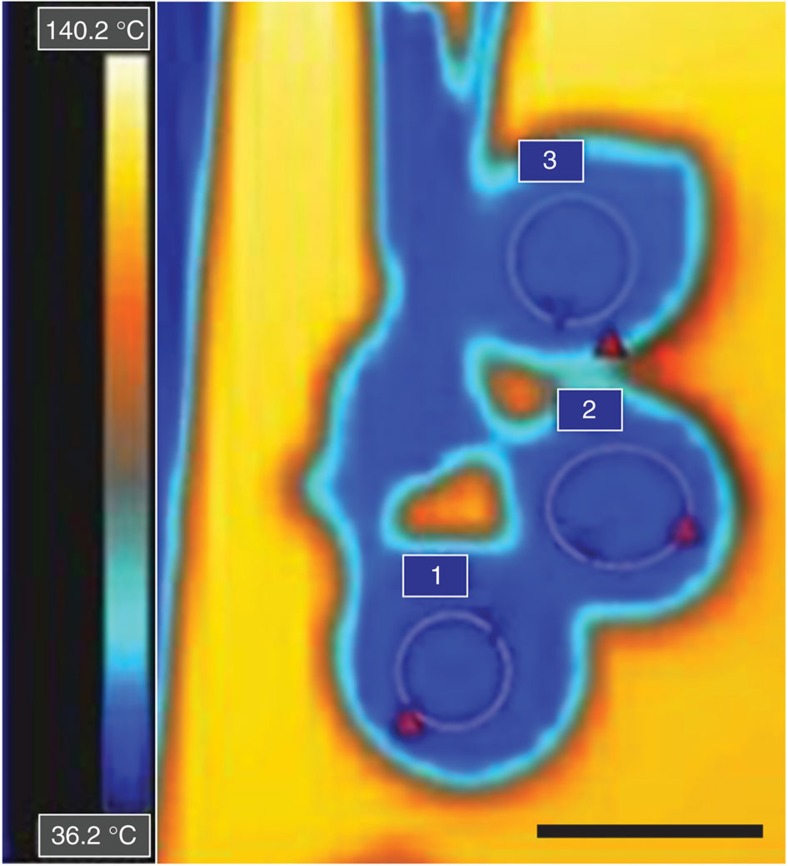
Infrared thermogram of DM fruit. Three DM fruit in an infructescence being monitored by the FLIR E60 infrared camera, with numbers for each fruit indicating the area of interest where measurements were made. In this example, no one fruit has started to show thermogenesis, but the change will eventually occur in the fruit labelled ‘2’, which will then become the test fruit; the fruit labelled ‘3’ did not explode for another 5 min, so it was considered the control. Scale bar, 3 mm.

Groups of ripe DM (*Arceuthobium americanum*) fruit (*n*=9 groups of 2–3 fruits) were collected from a stand of infected lodgepole pine (*Pinus contorta* var. *latifolia*) near Kamloops, BC (50°31′ latitude and 120°28′ longitude) in early September 2013, and placed into a chamber in which the heat was increased at a rate of ~2 °C min^−1^. A sample size of nine would readily be able to show real differences in temperature, as temperature anomalies should truly never occur randomly. Average surface temperatures of all DM fruit in the chamber (Fig. 4) were measured using a FLIR E60 infrared camera (thermal sensitivity: <0.05 °C, 2% accuracy) and recorded as a time series using FLIR ThermaCAM software (Version 4.0.13284.103). The fruit that exploded first was deemed the test fruit, and its time series was referred to as *T*_test_, whereas an adjacent fruit that did not explode within the same group in the chamber was called the control fruit, and its time series was termed *T*_control_. It is possible to use an unexploded fruit as a control, since fruit will explode at different temperatures and therefore at various times, presumably due to different states of ripeness. As will be shown, anomalous heating occurs 1–2 min before dehiscence, and any fruit chosen as a control exploded much later (>3 min) than the test fruit.

Since we wished to detect internal heating, the time series *T*_test_ was compared with *T*_control_. The *T*_test_*−T*_control_ data were plotted versus time. If no endogenous heat is generated, plots of *T*_test_−*T*_control_ should be constant over time. Endogenous heat production manifests as a positive deviation of the temperature difference. Confidence intervals for the temperature difference and trend lines were calculated using Excel 2007 (Microsoft).

### Modulated differential scanning calorimetry

A differential scanning calorimeter (2910 modulated refrigerated differential scanning calorimeter, New Castle, Delaware) was used to generate thermograms from DM fruit 17.0±3.1 mg (*n*=42, s.d.) collected from the infected pine stand (described in previous section) during the first week of September in both 2013 and 2014. Again, a bona fide heat increase should not be a random occurrence, so our sample size of 42 is large enough to detect genuine thermogenesis. MDSC provides total heat flow data that are separated into reversing and non-reversing events. Both non-isothermal and isothermal experiments were performed. Non-isothermal MDSC has been used to measure thermogenic energy release (for example, from cytoplasmic male sterile rice mitochondria)^14^, and isothermal MDSC has been similarly used to monitor non-reversible heat flows and exothermic events (for example, exotherms associated with polymer curing)^15^; thus, both types of MDSC will readily identify thermogenesis. For the non-isothermal experiments, samples were equilibrated at 5 °C and held under isothermal conditions for 10 min. The temperature was then ramped at 1.0 °C min^−1^ up to 65–80 °C with modulation±0.159 °C min^−1^. For the isothermal experiments, the samples were equilibrated at 25, 37 or 50 °C. The temperature was modulated ±0.159 °C min^−1^. The system was held under these conditions for 30–300 min. All data were analysed and integrated consistently using a sigmoidal baseline calculation^16^ to reveal trends and thermal events (TA Universal Analysis program, version 2000).

## Additional information

**How to cite this article**: deBruyn, R. A. J. *et al*. Thermogenesis-triggered seed dispersal in dwarf mistletoe. *Nat. Commun.* 6:6262 doi: 10.1038/ncomms7262 (2015).

## Figures and Tables

**Figure 1:**
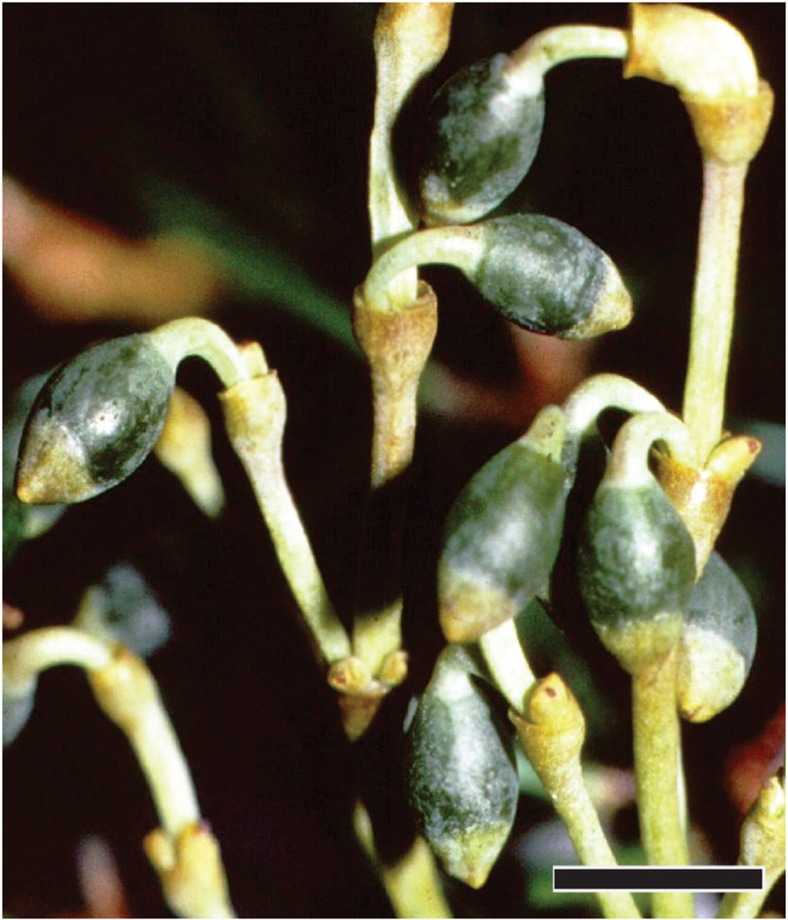
Ripe dwarf mistletoe (DM) fruit. DM fruit collected at the end of the growing season (early September in British Columbia) are broadly fusiform-spheric, bicoloured and borne on short, recurved pedicels in groups of three or more on branching infructescences. Discharge of the seed will ultimately occur at the abscission layer, located where the pedicel contacts the fruit. Scale bar, 4 mm. A version of this photograph was originally published in a paper by Kelly *et al*.^17^
