# Author Correction: Thermogenesis-triggered seed dispersal in dwarf mistletoe

**DOI:** 10.1038/ncomms16218

**Published:** 2018-06-25

**Authors:** Rolena A. J. deBruyn, Mark Paetkau, Kelly A. Ross, David V. Godfrey, John S. Church, Cynthia Ross Friedman

Nature Communications 6: Article number: 6262; DOI: 10.1038/ncomms7262 (2015); Published: 02 09 2015, Updated: 06 25 2018

John S. Church, who provided access to the FLIR E60 infrared camera and provided guidance in its use, was inadvertently omitted from the author list in the originally published version of this Article. This has now been corrected in both the PDF and HTML versions of the Article.

